# Expert Consensus on Telemedicine Management of Diabetes (2020 Edition)

**DOI:** 10.1155/2021/6643491

**Published:** 2021-03-24

**Authors:** Bo Zhang

**Affiliations:** Department of Endocrinology, China-Japan Friendship Hospital, Beijing 100029, China

## Abstract

Diabetes is a chronic disease that is very suitable for telemedicine management. Owing to the coronavirus disease 2019 (COVID-19) pandemic, telemedicine management of diabetes is particularly important. This consensus proposes 38 recommendations for key issues in telemedicine management of diabetes and provides preliminary specifications for diabetes management. It is recommended to use the most current information and communication technologies for telemanagement of patients' diet, exercise, behavior, and drug therapy. A strategy for drug therapy that is more suitable for telemedicine management of diabetes than previous ones is established. During online follow-up visits, major changes of antihyperglycemic agents must be avoided, and treatment modification should be done in a stepwise manner. Greater attention should be paid to the occurrence and prevention of hypoglycemia, in addition to educating patients about the side effects of the drugs used and encouraging them to actively report adverse drug reactions. Conditions are clarified under which online follow-up visits must be terminated and in-person visits or emergency visits must be initiated. Telemedicine can improve the management level and control rate of diabetes. The present consensus for the standardized diagnosis and treatment of diabetes can reduce the potential risks of telemedicine management, yield great benefits to patients, and reduce chronic complications and comorbidities.

## 1. Introduction

Diabetes is a chronic disease that seriously threatens the health of patients. Under the present management model, the control rate of diabetes is poor, and there is an urgent need for greater use of telemedicine, to improve the management level of this disease. Because the modification of treatment regimens for diabetes is only moderately challenging, diabetes is suitable for remote diagnosis and treatment, representing a chronic disease that is very amenable to telemedicine management. With the rapid development of Internet technology and constant health care reforms, the management model for patients with diabetes has gradually changed. The development of mobile applications (apps) and wearable devices provides new possibilities for medical organizations or specialist teams to use telemedicine management for patients with diabetes, including online diagnosis and treatment. In the context of the national policy “Internet + Health care,” medical insurance policies in China have driven online health care services in medical organizations and Internet hospitals, to enter a rapid development stage. The pandemic of coronavirus disease 2019 (COVID-19) has further advanced the telehealth management model.

However, the present telemedicine management of diabetes is in its initial stages and at a small scale, serving limited patient populations. Because telemedicine management of diabetes is still emerging, there are no guiding specifications to ensure its scientific basis and safety, especially regarding the determination of management limitations. Therefore, the Expert Committee on Diabetes of the National Telemedicine and Connected Health Care Center of China, in conjunction with the Endocrinology and Diabetes Society of Chinese Bethune Spirit Research Association, has brought together experts in endocrinology to compile an expert consensus on telemedicine management of diabetes. This consensus is based on national and international guidelines and consensuses as well as the latest research in diabetes-related fields, in consideration of the national conditions and relevant regulations in China. The present consensus is expected to provide preliminary specifications for the telemedicine management of diabetes.

## 2. Scenarios Suitable for Telemedicine Management of Diabetes

 
*Recommendation 1*. Medical organizations implementing telemanagement should be certified by the National Center for Chronic Disease Telemedicine Management and associated health departments, receive the corresponding quality control supervision, and have qualifications related to telemanagement. Medical services must comply with relevant laws and regulations (strong recommendation, C; Table 1).
 
*Recommendation 2*. Telemanagement teams should include, but are not limited to, practicing physicians in relevant specialties, diabetes education nurses with quality certifications, dietitians, and telemanagers (strong recommendation, C).
 
*Recommendation 3*. The telemanagement system can be used to conduct comprehensive assessment and regular monitoring of patients with diabetes and to promote early screening and early warning for diabetes and its complications (strong recommendation, C).
 
*Recommendation 4*. The telemanagement system can be used for health education, individualized visual dietary and exercise guidance, and efficacy assessment of patients with diabetes (strong recommendation, A).
 
*Recommendation 5*. The telemanagement system can be used to follow up patients with diabetes, including the management of blood glucose, blood pressure, blood lipids, and complications (recommendation, C).
 
*Recommendation 6*. The telemanagement system can be used to conduct online follow-up visits for patients with diabetes (strong recommendation, A).

Telemedicine management, as a subset of telehealth, mainly refers to a management model that adopts modern information and communication technologies to enable medical staff to intervene and guide patients with respect to their lifestyle and drug therapy in a timely manner and to help patients control and manage their illness, thereby providing remote medical services. Medical organizations implementing telemanagement should be certified by the National Center for Chronic Disease Telemedicine Management and associated health administrative departments with telemanagement-related qualifications. Medical teams for telemanagement should be integrated and should include endocrinologists, diabetes specialist nurses, dietitians, and professional computer managers. Normal operation and implementation of the diabetes telemanagement model is supported by technologies such as the Internet and remote monitoring. Previous research has been shown that monitoring and management of blood glucose levels and medications in patients with diabetes using a telemanagement system can remarkably improve patients' metabolic indices. The mobile app systems used in previous research include a blood glucose data management system, a dietary habit data management system, a physical activity data management system, a personal goal setting system, and a general diabetes information query system. Past research results have indicated that patients' dietary habits are clearly modified, and their physical activity frequency and intensity as well as self-management skills are substantially improved. To date, there have been very few studies conducted among patients with diabetes receiving telemanagement in China. In particular, the benefits for long-term clinical outcomes and health economic assessment lack sufficient evidence to support evidence-based medicine. Additionally, the app systems used for telemedicine management of diabetes need to be further developed and tested, as these may have greater applications in transitional care for specific populations. In general, randomized controlled trials involving larger sample sizes and longer time periods are needed to clarify the role of the telemanagement model in reducing long-term complications among patients with diabetes [1–3].

## 3. Diabetes Populations Suitable for Telemedicine Management

 
*Recommendation 7*. All patients with diabetes who are able to interact with telemanagers, accept individualized management, and sign informed consent forms can receive telemanagement through the web-based platform (recommendation, B).
 
*Recommendation 8*. The population with diabetes most suitable for telemanagement are patients with type 2 diabetes mellitus (T2DM) (strong recommendation, A).
 
*Recommendation 9*. Patients with mental illness, cognitive impairment, and acute diabetes complications should not receive telemanagement (strong recommendation, A).

Currently, telemedicine management of diabetes is an extension of the conventional management model. The core function of telemanagement is to implement long-term continuous monitoring of health indices including blood glucose, blood pressure, and body weight and to guide patients in changing their lifestyles. Therefore, telemanagement is presently a vital supplement to conventional diagnosis and treatment modalities. Telemanagement has the advantage of being immediate and efficient. On the one hand, medical staff analyze data and feed back guidance information using the telemanagement system; on the other hand, using the interactive telemanagement system platform, patients can fully interact in real time, deal with problems efficiently, and improve their compliance.

All patients with diabetes who are able to upload self-test data and receive educational information, as well as interact with telemanagers and receive individualized management, can use the web-based platform to receive telemanagement.

  Web-based telemanagement can save time and transportation costs for patients with various types of diabetes (T2DM, gestational diabetes mellitus, and type 1 diabetes mellitus) and can contribute to improving patients' healthy behavior, self-management ability, and treatment compliance [4]. Moreover, many studies have shown that compared with conventional management, telemanagement of T2DM can markedly improve glycated hemoglobin (HbA1c) levels; therefore, patients with T2DM are the population most suitable for telemanagement [5].
  Because telemanagement covers a wide range of areas, medical services can be provided for a greater number of patients, especially those with diabetes living in areas that have a shortage of health resources. Therefore, patients with diabetes in remote areas can participate in telemanagement as an important auxiliary means of chronic disease management.
  Patients with mental illness or cognitive impairment cannot effectively communicate and interact with telemanagers and are therefore not suitable for participation in telemanagement. Additionally, patients who have unstable conditions of diabetes, as evaluated by a health practitioner, are not suitable for participation in telemanagement. These patients are advised to improve their conditions via hospital visits.
  Currently, many studies on telemedicine have demonstrated the potential advantages and considerable value of diabetes telemanagement for glycemic control; however, the actual clinical applications of telemedicine are still limited and an evidence gap exists in this regard. Moreover, a large number of studies have been conducted for a short duration, only focusing on short-term improvement in blood glucose levels. It is suggested that further assessments be carried out in the future by focusing on improvements in long-term complications, mortality, quality of life, compliance, and satisfaction in patients with diabetes, as well as health economics. Furthermore, multicenter randomized controlled trials involving longer study periods and large sample sizes are required, to further explore the advantages and disadvantages of the diabetes telemanagement model, clarify the populations that would benefit most from telemanagement, and provide a reliable theoretical foundation for health care model reform.

## 4. Data Collection and Assessment in Telemedicine Management of Diabetes

 
*Recommendation 10*. It is suggested to perform a comprehensive offline assessment for first visits. At present, online diagnosis and treatment are only available for follow-up patients (strong recommendation, A).
 
*Recommendation 11*. During follow-up visits, the electronic medical record system can be used to confirm a patient's identity and collect complete information. Electronic medical records are standardized and the data are secure, with privacy protections in place (strong recommendation, C).
 
*Recommendation 12*. Physicians can complete part of the physical examination via video, such as checking skin lesions and diabetic foot ulcers; examination of the symmetry of gait and facial lesions; skin abnormalities at the insulin injection site; and goiter (strong recommendation, C).
 
*Recommendation 13*. The results of patients' self-monitoring or dynamic monitoring of blood glucose should be obtained and analyzed (strong recommendation, A).
 
*Recommendation 14*. It is recommended to perform a urine ketone test in the case of hyperglycemia, especially for patients with type 1 diabetes mellitus. Patients treated with SGLT-2i also should perform a urine ketone test (recommendation, C).
 
*Recommendation 15*. Tests required for a follow-up visit, such as HbA1C and urine albumin-to-creatinine (A/C) ratio, must be arranged. Time course for testing is 3–6 months for HbA1c and 12 months for A/C ratio, respectively (strong recommendation, C).
 
*Recommendation 16*. Referral to offline or emergency visits is required under the conditions listed in Table 2.

## 5. Education and Behavior Management

 
*Recommendation 17*. Diabetes tele-education can make full use of the advantages offered by telemedicine, to reduce the risks associated with telemanagement and should follow the rule of patient-centered, shared decision-making (recommendation, C).
  The telemanagement team for diabetes should include specialist physicians in endocrinology as well as exercise therapists, diabetes specialist nurses, clinical dietitians, professional computer managers, and telemanagement assistants [7]. It is suggested that [8] psychotherapists or psychiatrists be included on the team, to provide professional psychotherapy services if needed.
  The goal of diabetes self-management tele-education [9] is to support decision-making, modify self-management behavior, solve patients' problems, and encourage patients to actively cooperate with medical teams, thereby improving the clinical outcomes, health status, and quality of life in patients with diabetes.
  The appropriate times to provide diabetes tele-education [6, 10], so as to encompass both timely and long-term characteristics, include the following: at the time of diagnosis; during annual assessment and education; when new complex factors affect self-management; and in the case of hospital admission and discharge, or during transitional care, such as with the appearance of cognitive changes caused by age factors.
  The framework of diabetes tele-education should include the following: all patients with diabetes must undergo patient-centered assessment and referral. A combination of offline data collection and online teleassessment can be used to assess education targets including [7] disease condition, knowledge, behavior, and psychological aspects. For patients requiring treatment by a psychologist or dietary specialist, referral support should be provided after timely assessment.

The advantages of diabetes tele-education can benefit both patients and society as a whole. However, there is still a lack of evidence with respect to record keeping, data security, privacy protection, costs and payment, and risk management in diabetes tele-education. Building a more standardized and comprehensive diabetes tele-education system, with the assistance of multiple sources, is recommended.

## 6. Dietary Management

 
*Recommendation 18*. Dietary management should be performed for all patients with diabetes throughout the treatment period (strong recommendation, A).
 
*Recommendation 19*. Prior to initiating dietary management, patients with diabetes should undergo assessment of their nutritional status. Dietary prescriptions for patients with diabetes should be formulated following the principles of individualization, scientific evidence, and safety (strong recommendation, A).
 
*Recommendation 20*. Applications that have been proven effective in clinical trials can be used to manage diet (recommendation, B).

### 6.1. General Rules of Dietary Management

Dietary telemanagement for diabetes should incorporate telemonitoring and telemanagement of the diet in patients with diabetes, using computer and Internet technologies, mobile phones, and intelligent electronic devices, with coordination by a multidisciplinary team.

Before initiation of dietary management, the nutritional status of patients with diabetes should be assessed, including individualized nutritional assessment, diagnosis, and formulation of corresponding nutritional intervention plans. It is suggested to set reasonable nutritional therapy goals, adjust total energy intake, allocate various nutrients in a rational and balanced manner, and meet individual dietary preferences as much as possible.

### 6.2. Suggestions on Diet for Special Populations

Children and adolescent patients with T2DM should control their diet, to maintain a standard body weight, correct metabolic disorders, and reduce the burden on pancreatic *β* cells. Dietary consumption should total 900–1,200 kcal/d for children aged 6–12 years old and >1,200 kcal/d for adolescents aged 13–18 years old.

Diets for patients with type 1 diabetes mellitus should be even more individualized, comprising smaller meals eaten more often, to prevent the occurrence of hypoglycemia.

In older patients with diabetes, a comprehensive dietary assessment is required because of their relatively poor gastrointestinal function and greater complications.

Patients with gestational diabetes mellitus must ensure that the energy needs of both mother and fetus are met. Carbohydrates with a low glycemic index should be selected as much as possible, with smaller meals consumed more often (5–6 meals per day).

Patients with chronic diabetes complications should be given individualized dietary guidance and precautions.

### 6.3. Telemonitoring and Telemanagement

Patients should be encouraged to use intelligent telemanagement systems for timely feedback, and telemanagers should follow up with patients in a timely manner, to supervise and guide them in regimen implementation. The interactive telemanagement system platform can be used to promote interaction and information exchange between telemanagers and patients.

Patients should be instructed to perform self-monitoring of relevant indices before, during, and after dietary control, including blood glucose, blood lipids, blood pressure, and body weight.

Patients should be guided to complete dietary diaries using intelligent monitoring equipment that records dietary intake, exercise, medications, and blood glucose.

Tele-education and teletraining of patients should be carried out regularly.

### 6.4. Regular Assessment and Adjustment

Intelligent devices can be used to track dietary activities and provide patients with regular reminders.

Offline assessment of metabolism, physical fitness, and body shape index, as well as online quality of life and self-management behavior change assessments, should be conducted regularly. The diet plan should be implemented progressively and adjusted when appropriate.

## 7. Exercise Rehabilitation Management

 
*Recommendation 21*. Exercise rehabilitation management should be carried out for all patients with diabetes, after excluding those with contraindications for exercise, throughout the treatment period (strong recommendation, A).
 
*Recommendation 22*. Before initiating exercise rehabilitation, patients with diabetes should undergo preexercise assessment. Exercise prescriptions for patients with diabetes should be formulated following the principles of individualization, scientific evidence, and safety (strong recommendation, A).
 
*Recommendation 23*. For special diabetic populations, suggestions on exercise rehabilitation management should be provided (recommendation, C).
 
*Recommendation 24*. Intelligent applications that have proven effective in clinical trials can be used to manage exercise (recommendation, B).

### 7.1. Preexercise Assessment

Exclusion of contraindications for exercise: exercise is contraindicated in the case of fasting blood glucose >16.7 mmol/L, recurring hypoglycemia or blood glucose fluctuations, diabetic ketoacidosis, proliferative retinopathy, severe nephropathy, severe cardiocerebrovascular diseases (unstable angina, severe arrhythmia, and transient ischemic attack), and concomitant acute infections [11].

Required preexercise assessment: when asymptomatic individuals perform low-intensity physical activities that are no more strenuous than brisk walking or daily life activities during the early stages of exercise, it is not necessary to perform preexercise medical screening [12]; instead, online basic physical condition assessment and medical assessment can be carried out. If moderate- or high-intensity exercise is to be performed, it is necessary to visit a medical center for offline assessment and exercise testing, including physical fitness assessment (involving cardiorespiratory endurance, body composition, muscle strength, and flexibility), medical assessment, and nutritional assessment.

### 7.2. Suggestions for Exercise Rehabilitation in Patients with Diabetes

Adult patients with diabetes should perform at least 150 minutes of moderate-intensity aerobic exercise per week, over at least 3 days and no more than 2 consecutive days without exercising [12].

Adult patients with diabetes should perform two to three sessions of moderate-intensity resistance exercise per week that involves all major muscle groups; the same muscle group should not be trained on 2 consecutive days [12].

High-intensity interval training is suitable for young patients with certain levels of physical fitness, and it is recommended that these patients perform high-intensity interval training for more than 75 minutes a week [12, 13].

Exercise should be done within 1–3 hours after a meal. The training process should include 5–10 minutes of warm-up exercises, at least 10 minutes of effective exercises, and 5–10 minutes of relaxation exercises.

Daily sedentary time should be reduced, and it is recommended to interrupt sedentary activities with performance of some light physical activities every 30 minutes [13].

### 7.3. Suggestions on Exercise Rehabilitation Management for Special Populations with Diabetes

Children and adolescents with diabetes should be encouraged to engage in moderate- to high-intensity aerobic exercises at least 60 minutes per day, in addition to muscle strengthening and bone strengthening activities for at least 3 days per week [14].

The exercise type and duration for patients with type 1 diabetes mellitus should be adjusted individually, and blood glucose monitoring should be added, as appropriate, to prevent the occurrence of hypoglycemia.

Older patients with diabetes should engage in flexibility and balance training two to three times per week [12].

Patients with gestational diabetes mellitus are advised to engage in moderate physical activities [14].

Patients with concomitant chronic diabetes complications should be given individualized exercise guidance and precautionary advice.

Patients should undergo regular offline metabolism assessment, as well as assessment of physical fitness, body shape index, and exercise ability, online quality of life assessment, and self-management behavior change assessment. The exercise plan should be implemented progressively and adjusted as appropriate. Use of intelligent apps that have been proven effective in clinical trials is encouraged, to manage exercise. Wearable devices (such as pedometers and smart bracelets) can be used to provide valuable information for timely assessment and adjustment of exercise prescriptions.

## 8. Antihyperglycemic Treatment for T2DM

 
*Recommendation 25*. Drug therapy for diabetes should follow the principle of individualization, with drug selection based on clinical features and compliance of the patient (strong recommendation, A).
 
*Recommendation 26*. Artificial intelligence can be used to assist, rather than replace, practitioners in online diagnosis and treatment (strong recommendation, C).
 
*Recommendation 27*. All patients should be educated on hypoglycemia and the treatment methods (strong recommendation, C).
 
*Recommendation 28*. Major changes in the treatment regimen should be avoided via online diagnosis and treatment. Dose adjustment of all oral antihyperglycemic agents/injections must be done in a stepwise manner (strong recommendation, C).
 
*Recommendation 29*. Patients with diabetes who are eligible for telemanagement should first be assessed for the following factors: (1) whether they have concomitant atherosclerotic cardiovascular disease (ASCVD) or high cardiovascular risk factors (≥55 years old with one of the following conditions: coronary arterial, carotid, or lower extremity arterial stenosis >50% or left ventricular hypertrophy); (2) whether the patient belongs to a high-risk population for hypoglycemia; (3) whether they have concomitant heart failure (HF; especially heart failure with reduced ejection fraction, HFrEF); (4) whether the patient has concomitant chronic kidney disease (CKD); and (5) whether they have an urgent need to lose weight (strong recommendation, C).
 
*Recommendation 30*. It is recommended to use sodium-glucose cotransporter-2 inhibitors (SGLT-2is) and glucagon-like peptide-1 receptor agonists (GLP-1RAs) as single or combined use in patients with diabetes who have ASCVD or very high risk (strong recommendation, B).
 
*Recommendation 31*. Semaglutide and dulaglutide may have a protective effect against stroke whereas pioglitazone may have a protective effect against stroke, as secondary prevention (recommendation, B).
 
*Recommendation 32*. SGLT-2is are the first option for patients with T2DM and concomitant HF when there are no contraindications (strong recommendation, B).
 
*Recommendation 33*. SGLT-2is are the first option for patients with T2DM and concomitant CKD when there are no contraindications (strong recommendation, B).
 
*Recommendation 34*. The preferred option for patients with T2DM and a high risk of hypoglycemia is single or combined use of drugs that do not increase the risk of hypoglycemia, such as metformin, dipeptidyl peptidase-4 inhibitors (DPP-4is), *α*-glucosidase inhibitor, thiazolidinedione, SGLT-2is, or GLP-1RAs (recommendation, C).
 
*Recommendation 35*. Combined use of GLP-1RAs or SGLT-2is on the basis of metformin is preferred for patients with T2DM and concomitant obesity who are in urgent need of weight loss (recommendation, C).
 
*Recommendation 36*. Basal insulin can be used to control fasting blood glucose in combination with oral drugs, as it has a lower risk of hypoglycemia than other insulin treatment regimens and is more suitable for online management (recommendation, C).
 
*Recommendation 37*. Hospital visits are generally required when practitioners consider that there is a need for a transition of the insulin treatment regime (general recommendation, C).
 
*Recommendation 38*. Patients should be informed of common side effects of the antihyperglycemic agents used [15] (strong recommendation, C).

Drug therapy is the key component of telemedicine for diabetes management. At present, comprehensive management of ASCVD and CKD in T2DM, including antihyperglycemic, antihypertensive, lipid-lowering, and antiplatelet therapy, is of great importance. Recently, results from a series of large-scale clinical trials have shown that in addition to evidence of an antihyperglycemic effect, some newer antihyperglycemic agents have cardiovascular and renal benefits. Therefore, reasonable and standardized use of antihyperglycemic agents requires hierarchical management and selection of reasonable glycemic control regimens, based on the patients' disease condition and other factors such as age, disease course, complications, and risk of adverse drug reactions. This section focuses on telemanagement of blood glucose in T2DM, including cardiovascular risk assessment and classification, formulation of glycemic control targets, characteristics of first- and second-line antihyperglycemic agents, hierarchical management and implementation strategy, and recommended drugs.

### 8.1. Glycemic Control Targets in T2DM

The targets of HbA1c control should follow the principle of individualization, that is, hierarchical management should be implemented based on the patient's disease condition and other factors such as age, disease course, complications, and risks of adverse drug reactions, and scientific assessment of glycemic control should be conducted in terms of the risk-benefit ratio, benefit-cost ratio, and accessibility, to achieve the most reasonable balance. Specific details include the points outlined as follows:

  The target value of HbA1c is <7.0% for most adult patients with T2DM [8].
  The recommended control target of HbA1c is ≤6.5% or as close to normal as possible if patients have a relatively young age, short course of disease, long life expectancy, no complications, no concomitant CVD, and the antihyperglycemic agents used do not increase the risk of hypoglycemia [16].
  The recommended target value of HbA1c is ≤7.5% for older patients with a good health condition (few concomitant chronic diseases, good physical function, and good cognitive function), patients with CKD aged <40 years, patients with stage 1–2 CKD aged ≥40 years, and patients with stage 3–4 CKD who have not received insulin therapy [16].
  The recommended target value of HbA1c is between 7.0% and 8.0% in patients with T2DM who have a long disease course, concomitant ASCVD, or very high cardiovascular risk, older patients (age ≥60 years) who have moderately impaired health (with multiple concurrent chronic diseases, impaired ability of more than two daily life activities, or mild to moderate cognitive impairment) [16], and patients with T2DM and concomitant HF.
  The target value of HbA1c can be expanded to 7.5%–8.5% for patients with stage 3–4 CKD who are receiving insulin therapy or patients with stage 5 CKD who are undergoing hemodialysis [16].
  The target value of HbA1c can be further expanded to 8.0%–9.0% for older patients with poor health, patients with a high risk of hypoglycemia, patients with concomitant malignant tumor, Alzheimer disease, or epilepsy, and a life expectancy of <5 years, and patients who have difficulty implementing the treatment regime owing to mental or intellectual impairment or vision loss [16].

### 8.2. Drug Selection and Therapeutic Route for T2DM

**Figure 1:**
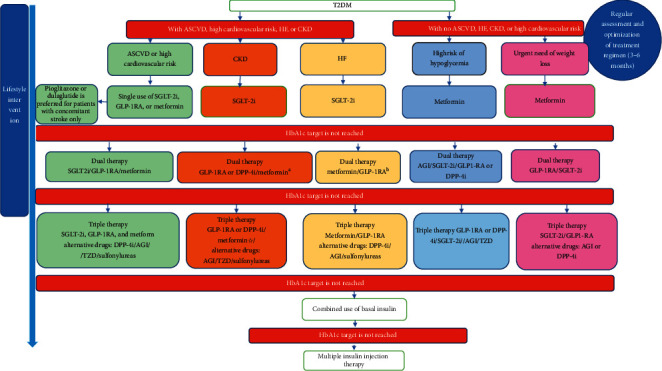
Flowchart of drug therapy for type 2 diabetes mellitus. T2DM: type 2 diabetes mellitus; ASCVD: atherosclerotic cardiovascular disease; HF: heart failure; CKD: chronic kidney disease; GLP-1RA: glucagon-like peptide-1 receptor agonist; SGLT-2i: sodium-glucose cotransporter-2 inhibitor; DPP-4i: dipeptidyl peptidase-4 inhibitor. (a) If patients have an estimated glomerular filtration rate (eGFR) <45 mL/min/1.73 m^2^, use of SGLT-2is and metformin is not recommended; it is suggested to select GLP-1RAs or DPP-4is, which have evidence of renal benefits. (b) For patients with T2DM who have HF, the use of SGLT-2is and metformin is not recommended with eGFR <45 mL/min/1.73 m^2^; it is preferable to use GLP-1RAs, which have evidence of cardiovascular benefit.

Nutrition and exercise therapies are the basic treatments to control hyperglycemia, and these should continue throughout all stages of treatment. When selecting antihyperglycemic agents, the principle of individualized treatment should be followed, and comprehensive patient assessment needs to be carried out in terms of comorbidities and complications, risk of hypoglycemia, impact on body weight, risk of adverse reactions, cost of treatment, and compliance of the patient (Figure 1).

#### 8.2.1. Recommendations for Preferred Drugs

  Metformin: this drug has an excellent antihyperglycemic effect, and it has no risk of hypoglycemia when used as a monotherapy [8, 15]. There is also rich experience in the clinical use of metformin. The medication has a low cost and may reduce the risk of cardiovascular death and all-cause death [17–19], offering multiple potential benefits beyond the antihyperglycemic effect. Metformin therapy is not recommended for patients with T2DM who have acute or severe HF. Renal insufficiency may lead to its accumulation in the body, thereby increasing the risk of lactic acidosis. The use of metformin therapy is not recommended in patients with an estimated glomerular filtration rate (eGFR) of <45 mL/min/1.73 m^2^.
  SGLT-2is: the major drugs in this group are empagliflozin, canagliflozin, and dapagliflozin. These can lower blood sugar, body weight, systolic blood pressure, and uric acid; regulate blood lipids and have a definite effect on cardiorenal protection [20–22]. Special attention should be paid to closely monitoring adverse drug reactions in patients with a high risk of genital infection, renal insufficiency, and hypovolemia.
  GLP-1RAs: the drugs in this group promote insulin secretion in a glucose-dependent manner, inhibit glucagon secretion, suppress appetite, and delay gastric emptying. The currently marketed drugs are short-acting exenatide, lixisenatide, and benaglutide; the long-acting preparation liraglutide; and weekly preparations including dulaglutide, exenatide, and loxenatide. GLP-1RAs should be injected subcutaneously, and monotherapy is associated with a low risk of hypoglycemia, with a role in reducing body weight, lowering systolic blood pressure, and improving blood lipids. Research results have shown that liraglutide, dulaglutide, albiglutide, and semaglutide injections can considerably reduce the risk of major adverse cardiovascular events (including cardiovascular death, nonfatal myocardial infarction, and stroke) [23–26]. The main adverse reactions are nausea, vomiting, and diarrhea.

#### 8.2.2. Second-Line Drugs

Because T2DM is a chronic progressive disease, combination therapy with multiple drugs is required with gradually declining function of islet cells. The following drugs can be selected: DPP-4is, insulin secretagogues, *α*-glycosidase inhibitors, thiazolidinediones, and insulin [7]. Insulin therapy is usually used in the final treatment regimen for cases with evident newly diagnosed symptoms, considerable elevation of HbA_1c_, or a long course of disease.

#### 8.2.3. Therapeutic Hierarchy of Glycemic Control for T2DM

  First, patients are divided hierarchically according to their disease condition: (1) whether they have concomitant ASCVD or high cardiovascular risk; (2) whether they have concomitant CKD (eGFR <60 mL/min/1.73 m^2^ or urine A/C > 30 mg/g, especially when urine A/C >300 mg/g); (3) whether they have concomitant HF; (4) whether they have a high risk of hypoglycemia; and (5) whether they have an urgent need to lose weight.
  Concomitant ASCVD or high cardiovascular risk: cardiovascular outcome trials (CVOTs) have demonstrated evidence of the cardiovascular benefits of SGLT-2is and GLP-1RAs in patients with T2DM who have ASCVD or high cardiovascular risk [20–24]. The United Kingdom Prospective Diabetes Study (UKPDS) demonstrated a possible cardiovascular protective effect of metformin. It is therefore recommended to select SGLT-2is, GLP-1RAs, or metformin, which have evidence of cardiovascular benefits, alone or in combination, for patients with T2DM or ASCVD or a high cardiovascular risk. If the target blood glucose level is not achieved, a combination with second-line drugs can be used. A meta-analysis revealed that the overall risk of stroke can be effectively reduced by up to 13% after the use of GLP-1RA therapy [27]. Dulaglutide reduces the risk of nonfatal stroke by 25% in patients with diabetes and high-risk factors for ASCVD; in particular, the incidence of ischemic stroke is reduced whereas there is no effect on hemorrhagic stroke [28]. Semaglutide reduces the risk of nonfatal stroke by 39% [25]. Another study showed that pioglitazone can reduce the risk of recurrence after ischemic stroke or transient cerebral ischemia [29]. The present consensus recommends that in the case of ASCVD, patients with diabetes and only stroke without coronary heart disease be given pioglitazone or dulaglutide as the preferred option; however, the cardiac function and fracture risk in these patients will need to be assessed when first using pioglitazone.
  Concomitant CKD: both renal outcome trials (ROTs; with renal outcomes as the primary endpoints) [30] and CVOTs (with renal outcomes as secondary endpoints) [20–22] have demonstrated that SGLT-2is can reduce urine albumin and improve renal “hard endpoints,” including a continuous decline in eGFR by ≥40%, progression to end-stage nephropathy, and death caused by nephropathy; therefore, this drug should be considered as the first option. The results of CVOTs indicate that GLP-1RAs can substantially reduce the risk of new-onset high-grade proteinuria [31–33]. If patients with CKD cannot use SGLT-2is, use of GLP-1Ras is recommended, which have evidence of renal benefits as a combination therapy, or to start DPP-4i therapy. Both linagliptin [34] and saxagliptin [35] have been shown to considerably reduce urine albumin and thus can be used as alternatives in combination therapy.
  Concomitant HF: CVOTs of SGLT-2is have consistently shown that these drugs can markedly reduce the risk of hospitalization owing to HF in patients with T2DM [20–22]. Thus, SGLT-2is should be the first option for these patients, as long as there are no contraindications. If the target blood glucose level is not reached after 3 months of treatment, metformin or GLP-1RAs can be used in combination therapy. If the target blood glucose level is not achieved, DPP-4is, *α*-glucosidase inhibitors, sulfonylureas, and insulin can be selected. It is not recommended to use thiazolidinediones or saxagliptin.
  Populations with a high risk of concomitant hypoglycemia: special attention should be paid to the safety of antihyperglycemic treatment in telemanagement. For patients with a higher risk of hypoglycemia or who experience greater effects owing to hypoglycemia (e.g., older people living alone), the preferred option is single or combined use of drugs that will not increase the risk of hypoglycemia, such as metformin, DPP-4is, *α*-glucosidase inhibitors, thiazolidinediones, SGLT-2is, or GLP-1RAs.
  Concomitant obesity with an urgent need to lose weight: combined use of GLP-1RAs or SGLT-2is on the basis of metformin is preferred. If there are contraindications or intolerance, the following drugs can be used: acarbose, which may partially reduce body weight [36], or DPP-4is, which have little effect on body weight.

If a combination of drugs still fails to achieve the target level of blood glucose in the abovementioned populations, combined insulin therapy can be considered. Because of higher safety requirements for telemanagement, it is suggested to first start using basal insulin, which has a relatively low risk of hypoglycemia and adjust the dosage according to fasting blood glucose. If the target blood glucose level is not reached, the patient can add mealtime insulin or change to premixed insulin. Patients should be advised to visit a hospital at the time of treatment regimen change.

#### 8.2.4. List of Recommended Drugs for Telemanagement

Preferred drugs are those with sufficient evidence for evidence-based medicine, those that are covered by medical insurance, and those showing good safety, considering both ease of accessibility and economic burden. To facilitate use in clinical practice, use of one to two drugs from each class is recommended (Table 3).

## 9. Conclusion

Under the background of the “Internet +” era and prevention and control during the COVID-19 pandemic, telemedicine-based antihyperglycemic treatment for T2DM can yield long-term benefits for disease status assessment, reaching targets via early glycemic control, and long-term maintenance. During selection of antihyperglycemic agents, the correct treatment regimen should be chosen by dividing patients into hierarchies and following the principle of individualized treatment. Comprehensive consideration is needed of prevention and control strategies for primary outcomes such as ASCVD, CKD, and HF while also paying attention to other factors such as antihyperglycemic efficacy, safety, compliance, and health economics. Glycemic control can be achieved via telemanagement according to the treatment route.

## Figures and Tables

**Table 1:** Definitions for the class of recommendation and level of evidence in this consensus.

Class of recommendation		Level of evidence	
Strong recommendation	>90% agreement of expert opinions	A	At least one randomized controlled trial or one high-quality meta-analysis
Recommendation	70–90% agreement of expert opinions	B	At least one randomized observational study, specific subgroup analysis, and large-scale observational study
Moderate recommendation	50–70% agreement of expert opinions	C	Nonrandomized controlled trials or expert opinions
No recommendation	<50% of expert opinions		

**Table 2:** Conditions that require referral for an offline or emergency visit.

Item	Content	Grade of recommendation and level of evidence
Hypoglycemia	Recurring hypoglycemia: random blood glucose <3.9 mmol/L, associated with other symptoms such as hunger, cold and clammy extremities, palpitation, and sweating	Strong recommendation, C
	Severe hypoglycemia: random blood glucose <2.8 mmol/L, irrespective of symptoms or not	
Diabetic ketoacidosis or hyperglycemic state	Dry mouth, polydipsia, nausea, vomiting, hyperpnea, and consciousness disorders	Strong recommendation, C
	Two or more times of fasting blood glucose ≥16.7 mmol/L or random blood glucose >20 mmol/L	
Chronic diabetic complications requiring emergency treatment [6]	(1) Patients having difficulties in online visit for diagnosis, treatment regimen formulation, and efficacy assessment of chronic diabetic complications (retinopathy, nephropathy, neuropathy, diabetic foot, or peripheral vascular disease)	Strong recommendation, C
	(2) Patients requiring emergency aid and treatment due to severe target organ damage caused by chronic diabetic complications (acute cardiocerebrovascular disease, renal insufficiency caused by diabetic nephropathy, severe vision loss caused by diabetic retinopathy, intermittent claudication and ischemic symptoms caused by diabetic peripheral vascular disease, and diabetic foot)	
Other stress conditions	Infection, fracture, trauma, foot ulcer, edema (continuous nonremission), blindness, cardiocerebrovascular emergency, and coma	Strong recommendation, C

**Table 3:** List of drugs recommended by the consensus for telemedicine management of diabetes.

Drug classification	Type of action	Drug varieties	Reason for recommendation
Injections	Short-acting insulin	Recombinant human insulin, biosynthetic human insulin	Cheap price
		Insulin aspart, insulin lispro	Low risk of hypoglycemia
	Intermediate-acting insulin	Protamine zinc recombinant human insulin, protamine biosynthetic human insulin	Cheap price
	Premixed insulin	Protamine recombinant human insulin (30/70), protamine biosynthetic human insulin (premixed 30R)	Cheap price
		Insulin aspart 30, protamine zinc recombinant insulin lispro (50R)	Low risk of hypoglycemia
	Long-acting insulin analogue	Insulin glargine	Included in the National Essential Medicines List of China
		Insulin degludec	A novel ultra-long-acting insulin with low risk of hypoglycemia [37].
	GLP-1RA	Liraglutide	It is included in the National Essential Medicines List of China and is the GLP-1RA with the strongest evidence of cardiovascular benefit.
		Dulaglutide	A weekly preparation, with evidence of cardiovascular and renal benefits [24, 32].

Oral antihyperglycemic agents	Biguanides	Metformin	
	Sulfonylureas	Glimepiride	A new generation of sulfonylureas with low risk of hypoglycemia; research has shown its cardiovascular safety similar to that of DPP-4i [38].
		Gliclazide sustained-release tablets	ADVANCE research suggests that gliclazide sustained-release tablets may have a renal protection; there is a low risk of hypoglycemia.
	Glinides	Repaglinide	It can be used throughout the course of CKD, with no need to adjust the dose.
	*α*-Glucosidase inhibitors	Acarbose	Widely used in the Chinese population [36], with cheap price.
	DPP-4i	Linagliptin	It can be used throughout the course of impaired hepatic and renal function, with no need to adjust the dose.
		Alogliptin	Meta-analysis reveals its good effect in Asian populations [39]; it has high affinity for DPP-4 and high bioavailability.
	SGLT-2i	Empagliflozin	It has definite benefits for ASCVD, HF, and CKD [22], and nowadays, it is the only marketed drug to reduce the risk of death from CVD.
		Dapagliflozin	It is included in the National Essential Medicines List of China. This drug has definite benefits for both HF and CKD [21], but there is insufficient evidence to determine whether it has benefit for ASCVD.
	Thiazolidinediones	Pioglitazone	PROACTIVE has found that it can reduce the risk of stroke recurrence [29].

GLP-1RA: glucagon-like peptide-1 receptor agonist; SGLT-2i: sodium-glucose cotransporter-2 inhibitor; DPP-4i: dipeptidyl peptidase-4 inhibitor; T2DM: type 2 diabetes mellitus; ASCVD: atherosclerotic cardiovascular disease; HF: heart failure; CKD: chronic kidney disease.
